# Efficacy of abdominal acupuncture for neck pain: A randomized controlled trial

**DOI:** 10.1371/journal.pone.0181360

**Published:** 2017-07-17

**Authors:** Lai Fun Ho, Zhi Xiu Lin, Albert Wing Nang Leung, Liyi Chen, Hongwei Zhang, Bacon Fung Leung Ng, Eric Tat Chi Ziea, Yuanqi Guo

**Affiliations:** 1 Chinese Medicine Services, Pok Oi Hospital, Hong Kong SAR, China; 2 School of Chinese Medicine, Faculty of Medicine, The Chinese University of Hong Kong, Hong Kong SAR, China; 3 Chinese Medicine Department, Hospital Authority, Hong Kong SAR, China; Stavanger University Hospital, NORWAY

## Abstract

**Objective:**

This study aims to provide evidence regarding the clinical efficacy of abdominal acupuncture for neck pain.

**Methods:**

This randomized, patient and assessor-blind, sham-controlled trial was conducted at a Chinese medicine center in Hong Kong between November 2014 and March 2016. A total of 154 eligible participants (age range, 18–65 years) with neck pain were randomly assigned to receive abdominal (n = 77) or non-penetrating sham abdominal (sham group; n = 77) acupuncture. Each participant was administered treatment over six sessions by Registered Chinese Medicine Practitioners, in accordance with a standardized protocol. The primary outcome was mean improvement in neck pain disability scores evaluated by the Northwick Park Neck Pain Questionnaire (NPQ). Secondary outcomes included intensity of neck pain and health-related quality-of-life measures. The outcomes were assessed at baseline and at 2 and 6 weeks from baseline. Patients in the abdominal acupuncture group received additional follow-up evaluation at 14 weeks from baseline. Outcomes were evaluated by intention-to-treat analysis.

**Results:**

All participants provided informed consent for treatment and follow-up evaluation. Patients who received abdominal acupuncture exhibited greater improvement in NPQ scores than those who received sham treatment at both 2 and 6 weeks from baseline (intergroup mean differences, -5.75; 95% confidence interval [CI], -9.48 to -2.03; *P* = 0.008 and -8.65; 95% CI, -12.13 to -5.16; *P* < 0.001, respectively). The improvement in NPQ scores in the abdominal acupuncture group was even more significant at 14 weeks from baseline. Patients in the abdominal acupuncture group also exhibited significantly greater improvements in intensity of neck pain and a few quality-of-life measures than those in the sham abdominal acupuncture group, without any serious adverse events.

**Conclusion:**

These findings suggest that abdominal acupuncture is an effective alternative treatment for neck pain.

**Trial registration:**

Chinese Clinical Trial Registry ChiCTR-TRC-14004932.

## Introduction

Neck pain is a common and challenging health problem worldwide, with an average prevalence of 48.5% in the lifetime of an individual [1]. Neck pain affects people of all ages, with 12-month prevalence ranges of 12.1–71.5% and 34.5–71.5% among adults and children, respectively [2]. The condition is highly prevalent in Hong Kong, where it has an increasing impact [3–5]. The 12-month prevalence of neck pain among the population in Hong Kong is 64.6%, with approximately 37.8% of patients with neck pain suffering from moderate to severe pain [5]. Neck pain can affect the working ability as well as social life of individuals; approximately 18.9% and 13.7% of patients with neck pain experience work and social life-associated limitations, respectively, with approximately 25.2% of these patients having to consult health practitioners or seek complementary therapies, including acupuncture [5].

According to the Bone and Joint Decade 2000–2010 Task Force on Neck Pain and Its Associated Disorders, neck pain is specific to pain located in the anatomic region of the neck, with or without radiation to the head, trunk, or upper limbs [6]. Patients with neck pain have various invasive and noninvasive treatment options, including medication, injection, manual therapy, physical modalities, complementary and alternative medicine, education or advice, and surgery [7,8]. However, because of the paucity of clinical evidence from primary studies, the efficacies of currently available treatments for neck pain remain unclear [9–11].

Abdominal acupuncture is used in treatment of various disorders—especially pain and neurological disorders [12–15]. The results of two systematic reviews on randomized controlled trials (RCTs) suggested that abdominal acupuncture might be effective for treatment of neck pain; however, because of the lack of high quality and well-designed studies, the authors of the two reviews concluded that evidence regarding the efficacy of abdominal acupuncture was inconclusive [16,17]. Therefore, we conducted an RCT with the aim of evaluating the efficacy of abdominal acupuncture for treatment of neck pain.

## Methods

### Study design

This research work was driven by a patient and assessor-blind, sham-controlled, parallel comparison trial with randomization for comparison of efficacies of standardized abdominal acupuncture and non-penetrating sham abdominal acupuncture (sham group) in treatment of neck pain. The study was performed at Pok Oi Hospital—The Chinese University of Hong Kong Chinese Medicine Centre for Training and Research (Shatin), Hong Kong between November 2014 and March 2016. The entire range of follow-up was from November 25, 2014 until March 1, 2016. All participants signed consent forms prior to participation, although none received financial incentives. The study protocol was approved by the Joint Chinese University of Hong Kong—New Territories East Cluster Clinical Research Ethics Review Committee, Hong Kong (CRE-2013.627-T). The trial was registered with the Chinese Clinical Trial Registry (ChiCTR; www.chictr.org.cn; ChiCTR-TRC-14004932). The protocol for this trial, supporting Consolidated Standards of Reporting Trials [18] and STandards for Reporting Interventions in Clinical Trials of Acupuncture [19] checklists are available as supporting information; see S1 Protocol, S1 and S2 Checklists, respectively.

### Participants and setting

**Fig 1:**
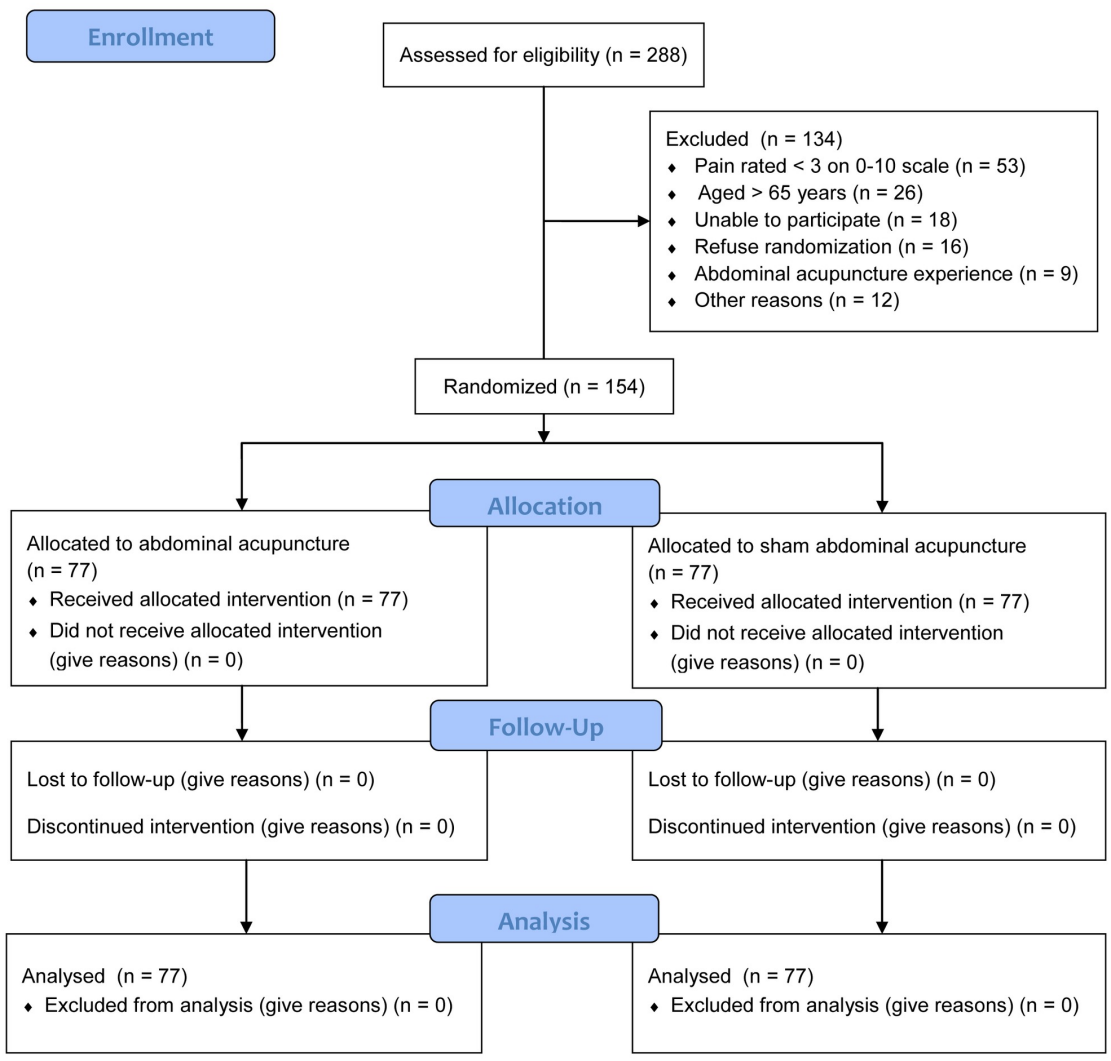
Study flow chart in accordance with the Consolidated Standards of Reporting Trials statement.

Participants were recruited from November 2014 to November 2015 through advertisement in media, posts on bulletin boards at all Chinese medicine centers, polyclinics, and mobile clinics under the management of Pok Oi Hospital, and leaflets delivered through these sites. Registered Chinese Medicine Practitioners (RCMPs) in Hong Kong with at least 3 years of clinical experience were trained by investigators to prescreen potential participants. Participants were considered eligible upon meeting the following conditions: (1) 18 to 65 years of age and frustrated with neck complaints including (a) pain, stiffness, or tenderness; (b) pain around the neck, with radiation towards the occiput or shoulders, or range of motion limited by neck pain; and (c) neck pain with degenerative joint disease or cervical spondylosis or both [20]; (2) visual analogue scale (VAS) score ≥ 3 points (10-cm scale) for neck pain intensity during screening; (3) no history of abdominal acupuncture; and (4) consent for random allocation and willingness to sign the informed consent form. Participants were excluded for the following reasons: (1) illness due to visceral pain in the neck, serious spinal disorders, or history of neck surgery or plan to undergo neck surgery during the study period; (2) chronic diseases that could interfere with the efficacy of abdominal acupuncture; (3) diagnosis of cancer of any nature; (4) chief pain complaint other than neck pain; (5) unsafe conditions for abdominal acupuncture or abdominal scars affecting the proper selection of acupuncture points; (6) severe psychiatric or psychological disorders; (7) acupuncture treatment within a month prior to this study, with conflicting or ongoing co-interventions; (8) participation in other clinical trials during this study; (9) pending neck-related litigation or disability claims; (10) inability to answer questionnaires and non-responsiveness towards the assessor; and (11) pregnancy and breast feeding. Fig 1 presents the flow chart of participant selection throughout the trial.

### Randomization and masking

Eligible participants were randomly assigned to the abdominal or sham abdominal acupuncture groups in a 1:1 ratio by computation [21] based on a block design, where the block size was not constant. Randomization was performed by an independent administrator who had no other role in this study. Allocation concealment was achieved by using sequentially numbered, opaque, sealed envelopes contained randomized assignments. All other individuals involved in the study—including patients, practitioners, and outcome assessors—were blinded to the randomization procedure. Participants and outcome assessors were also blinded to treatment allocation.

### Intervention

Half of the study participants received abdominal acupuncture—a therapeutic microsystem based on the traditional Chinese acupuncture meridian theory and new concepts developed by its inventor Dr. Zhiyun Bo [12–15,22]. Five RCMPs with an average of 12 years of clinical experience in acupuncture delivered treatment in accordance with the study protocol. One investigator was responsible for training the RCMPs in the standardized study procedures and for monitoring protocol compliance. All participants received 30-minute standardized treatments three times a week for two weeks (six treatment sessions in total).

**Fig 2:**
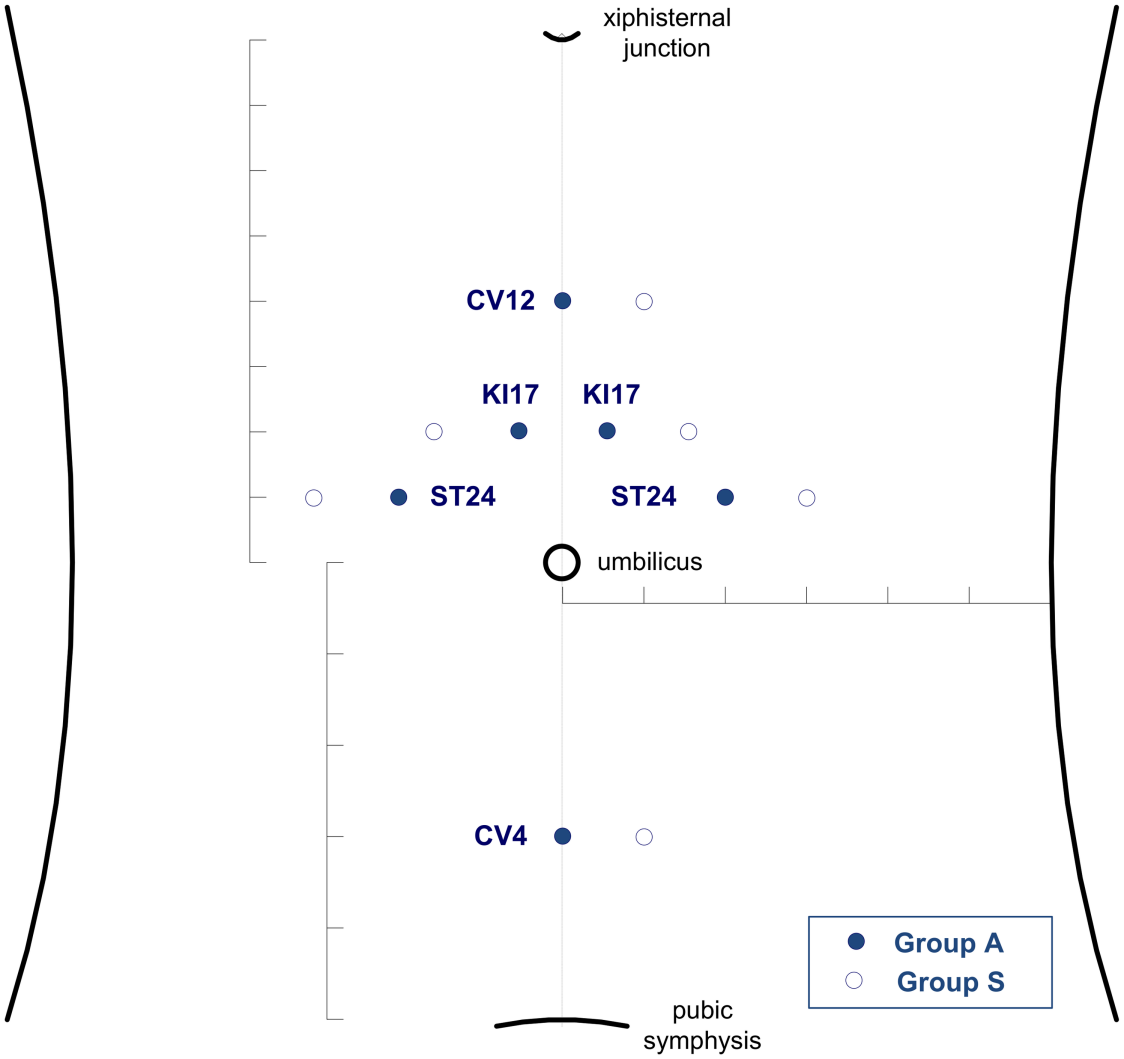
Acupuncture points used in this study.

Patients in the abdominal acupuncture group, designated as “group A”, received treatment in accordance with a predefined standardized abdominal acupuncture prescription described in previous studies on neck pain [12,23,24]. The following acupuncture points were stimulated: Zhongwan (CV12), Guanyuan (CV4), bilateral Shangqu (KI17), and bilateral Huaroumen (ST24) (Fig 2). The locations of these acupuncture points were determined on the basis of the guidelines presented in the World Health Organization Standard Acupuncture Point Locations in the Western Pacific Region [25]. Abdominal acupuncture was administered using sterile, single-use, disposable Bo’s abdominal acupuncture needles (size, 40 x 0.22 mm; Changzi City Enyi Science and Technology Co., Ltd, Beijing, China) with guide tubes for needle insertion. The tube rested on a plastic support with a hole in the center and an adhesive base. During treatment, the participants wore a black-eye-mask and lay down supine, with the abdomen exposed. The abdomen was first examined for any contraindication to abdominal acupuncture, and the skin was sterilized before needle insertion. Each treatment session began with needle insertion at acupuncture points CV12 and CV4, followed by insertion at bilateral KI17 and ST24. After needle insertion, the guide tubes were removed, while the adhesive plastic supports were retained on the chosen acupuncture points throughout the treatment process. Each abdominal acupuncture treatment session included three steps. The first step involved insertion of needles perpendicular to the superficial level of the skin at all of the selected acupuncture points. After 3 to 5 min, the second step was achieved by deep needling up to the superficial level of the abdominal muscle (depth, 20–35 mm) at CV12 and CV4, medium needling up to the subcutaneous abdominal fat (depth, 10–20 mm) at bilateral ST24, and shallow needling up to the superficial layer of the abdomen (depth, 5 mm) at bilateral KI17. Following this, the third step was achieved by making appropriate adjustments to the depths of needles based on the extent of pain relief experienced by the participant. The manipulation method was light, with only minimal twirling allowed. Responses regarding feeling of soreness, numbness, distension, heaviness, or muscle twitching were not sought from patients during treatment. The needles were retained for 30 min, with an infrared therapeutic lamp (Chongqing Xinfeng Medical Instruments Co., Ltd, Chongqing, China) placed 30 cm directly above the navel. Finally, the needles, along with the plastic supports, were removed following the sequence of needle insertion, with each insertion hole pressed down upon for a while.

Patients in the sham group, designated as “group S”, received treatment at non-acupuncture points 1 *cun* away from the acupuncture points stimulated in group A (Fig 2), with sterile, single-use, non-penetrating blunt sham needles (size, 40 x 0.30 mm; Suzhou Shenlong Medical Apparatus Co., Ltd, Suzhou, China) with guide tubes. During treatment, the participants wore a black-eye-mask and lay down supine, with the abdomen exposed. All treatment procedures and techniques—including inspection of the abdomen, skin sterilization, needle insertion, manipulation, retention, and withdrawal—were exactly the same as those applied in group A. The RCMPs pretended to insert and manipulate the blunt needles in a similar manner as that in group A. When the needle pressed against the skin, the blunt tip touched the skin, giving patients the feeling of actual needle insertion and manipulation. The guide tubes and adhesive plastic supports held the sham needles in place on the abdomen during treatment. An infrared therapeutic lamp was also placed 30 cm directly above the navel during the 30-minute needle retention time. Finally, the blunt needles, along with the guide tubes and plastic supports, were removed in a similar manner as that in group A. For ethical reasons, participants in this group were offered real abdominal acupuncture treatment after completion of the post-treatment assessment at 6 weeks from baseline.

During the investigative period, including the follow-up period, participants were advised by the RCMPs to avoid other treatments such as physiotherapy, acupressure, cupping, tuina, reflexotherapy, chiropractic, or bone-setting for neck pain. This instruction was reinforced by the research assistant during the baseline interview and at each evaluation time point. However, all study participants were allowed general light exercises.

### Outcomes

The primary outcome measure was the Northwick Park Neck Pain Questionnaire (NPQ) score [26]. The NPQ is used for evaluation of functional performance in patients with neck pain [27]. This study employed the validated Chinese version of the NPQ [28–30], which consists of nine five-part questions on activities that are likely to be affected by neck pain, for assessment of symptom severity. Each question is scored from 0 to 4 [26]. Participants were encouraged to answer all questions except the ninth one, which is a question addressed to people who drive cars. The NPQ scores have maximum values of 36 for car drivers and 32 for non-car drivers and are presented as percentages ranging from 0 to 100% [26,31], with higher scores indicating greater disability. For both groups, the NPQ scores were recorded at baseline, 2 weeks from baseline (upon completion of the sixth treatment session), and at 6 weeks from baseline (4-week follow-up after completion of treatments).

Secondary outcome measures included VAS scores for neck pain intensity and Short Form 36 Health Survey (SF-36) scores. The VAS for pain is a 10-cm continuous scale for measurement of pain intensity [27,32]. Participants were asked to indicate on the scale the degree of intensity of neck pain experienced on the day of assessment, with 0 indicating no pain and 10 indicating the worst imaginable pain. The SF-36 is a generic questionnaire used for evaluation of general health status, including physical and mental components, with higher scores indicating better health [27,33]. This study employed the validated Chinese (HK) SF-36v2 Health Survey [34]. For both groups, both secondary outcome measures were assessed at baseline and at 2 and 6 weeks from baseline. In addition, the pre-post first treatment VAS scores for neck pain were recorded in both groups.

For long-term evaluation of the effects of abdominal acupuncture in the active treatment group (group A), a subsidiary assessment was performed for measurement of all primary and secondary outcomes at 14 weeks from baseline (12-week follow-up after completion of treatments).

Treatment credibility in both groups was assessed using the well-validated credibility scale developed by Borkovec and Nau [35], which has previously been employed in acupuncture studies [33,36,37]. The credibility scale was used to assess the confidence of participants in this treatment alleviating their complaint, their confidence in recommending this treatment to friends with similar complaints, their perception of the logic of the treatment, and the likelihood of the treatment alleviating other complaints. These credibility scores—scored on a 7-point Likert scale (0 to 6 points), with higher scores indicating greater credibility—were recorded at baseline and at 2 weeks from baseline [33].

Upon completion of treatment at 2 weeks, all patients were evaluated for the global measure of satisfaction with care and treatment outcomes [38]. The efficacy of the blinding technique [39] in this trial was evaluated after the first treatment session and at 2 weeks from baseline. The number of participants who used pain-relief medications during the study period, acupuncture-associated complaints or adverse events, and dropout and attrition rates during the course of the investigation were recorded.

### Statistical analysis

Sample size was determined with the general acceptance of a 0.05-probability of a type I error and 80% power. Considering equal allocation of participants for the two treatment arms (abdominal acupuncture and sham abdominal acupuncture groups), sample size was determined assuming a repeated-measures of analysis of variance (ANOVA) using the statistical power analysis program G*Power 3 [40], with an effect size of 0.2, correlation among iterant measures of 0.5, and attrition rate of 15%. The results indicated that each group required 77 subjects, which implied a total sample size of 154 participants for the entire trial.

Continuous variables were summarized using means, standard deviations (SDs), and 95% confidence intervals (CIs) and categorical variables were summarized using counts and percentages. Data regarding demographic, clinical, and baseline characteristics and adverse events were analyzed descriptively and computed separately for each group. Baseline characteristics of both groups were assessed for differences. Intergroup differences in quantitative and qualitative data at baseline were evaluated by the independent two-sample t-test and Pearson chi-square test, respectively.

The effects of intervention over time for primary or secondary outcomes were assessed using the repeated-measures ANOVA model group (Group A and Group S) by time (baseline, 2 weeks from baseline, and 6 weeks from baseline) interaction. The Greenhouse-Geisser correction was applied upon violation of the assumption of Mauchly’s test of sphericity. For measures that indicated significant group by time interaction effects, post hoc analysis on differences between groups A and S were assessed by the independent sample t-test with Bonferroni correction; additionally, further analysis was performed using one-way repeated-measures ANOVA model including the 14-week follow-up data of group A.

Furthermore, comparison between Groups A and S for credibility assessment and blinding assessment were evaluated by the independent two-sample t test and Pearson chi-square test, respectively.

The scores of each domain of the SF-36v2 were computed using the Health Outcomes Scoring software version 4.5 (QualityMetric Incorporated, Lincoln, RI, USA). All statistical analyses were performed with the intention-to-treat approach with no missing data. Since all participants completed the treatments originally allocated, it was equivalent to a per-protocol analysis. The Statistical Package for Social Sciences software version 22.0 (IBM Corp, Armonk, NY, USA) was used for all statistical analyses. All tests were two-sided, and *P* values < 0.05 were considered statistically significant.

## Results

Of the 288 individuals who were initially recruited for this trial, 154 were found eligible and were randomly assigned to group A (n = 77) or S (n = 77). Participants were excluded from this trial for the following reasons: pain score < 3 points on a 10-cm VAS scale (n = 53), age > 65 years (n = 26), inability to attend treatment sessions (n = 18), refusal for randomization (n = 16), history of abdominal acupuncture (n = 9), and other reasons (n = 12). All participants attended six treatment sessions over a 2-week period and underwent all follow-up evaluations, with no data missing (Fig 1).

At baseline, there were no significant differences in sociodemographic characteristics and neck pain-related data between the two groups (Table 1). All participants were Chinese. The mean age and duration of neck pain were 45.02 ± 9.10 years (age range, 20–62 years) and 6.02 ± 5.56 years (range, 3 months to 30 years), respectively. Of the 154 participants, 23 (14.9%) reported taking pain-relief medications in the week prior to treatment. The mean NPQ and VAS scores were 41.13 ± 14.14 and 6.25 ± 1.65, respectively. The baseline data of all outcome measures in both groups exhibited homogeneity (Tables 2 and 3).

**Table 1:** Baseline characteristics of the participants.

	All (n = 154)	Abdominal Acupuncture (n = 77)	Sham Abdominal Acupuncture (n = 77)	*P* Value^g^
Sociodemographic characteristics				
Age, mean years (SD)	45.02 (9.10)	45.53 (8.74)	44.51 (9.48)	0.49
Female, n (%)	125 (81.2)	64 (83.1)	61 (79.2)	0.54
Education, n (%)^a^				0.18
≤ Primary school education	6 (3.9)	5 (6.5)	1 (1.3)	
Secondary school education	77 (50.0)	35 (45.5)	42 (54.5)	
Post-secondary education	71 (46.1)	37 (48.1)	34 (44.2)	
Acupuncture experience, n (%)^b^	83 (53.9)	42 (54.5)	41 (53.2)	0.87
History of neck pain				
Pain duration, mean years (SD)	6.02 (5.56)	6.04 (5.34)	6.01 (5.80)	0.97
Pain site, n (%)				
Neck only	3 (1.9)	1 (1.3)	2 (2.6)	0.56
Neck, with radiation to the occiput	53 (34.4)	27 (35.1)	26 (33.8)	0.87
Neck, with radiation to the shoulders	138 (89.6)	70 (90.9)	68 (88.3)	0.60
Neck, with radiation to the upper limbs	61 (39.6)	34 (44.2)	27 (35.1)	0.25
Pain occurrence, n (%)				0.87
Continuous	71 (46.1)	36 (46.8)	35 (45.5)	
Recurring	83 (53.9)	41 (53.2)	42 (54.5)	
Cervical radiography findings, n (%)				
Normal	33 (21.4)	17 (22.1)	16 (20.8)	0.84
Cervical lordosis abnormality	78 (50.6)	39 (50.6)	39 (50.6)	1.00
Narrowing of disc space	80 (51.9)	38 (49.4)	42 (54.5)	0.52
Other degenerative changes	87 (56.5)	48 (62.3)	39 (50.6)	0.14
Use of medications, n (%)^c^	23 (14.9)	10 (13.0)	13 (16.9)	0.50
Past treatment, n (%)^d^				
Western medicine	51 (33.1)	26 (33.8)	25 (32.5)	0.86
Chinese medicine	24 (15.6)	14 (18.2)	10 (13.0)	0.37
Acupuncture^e^	64 (41.6)	34 (44.2)	30 (39.0)	0.51
Physiotherapy	58 (37.7)	33 (42.9)	25 (32.5)	0.18
No treatment	21 (13.6)	7 (9.1)	14 (18.2)	0.10
Massage/Tuina	25 (16.2)	14 (18.2)	11 (14.3)	0.51
Pain-relief cream/oil/patch	19 (12.3)	10 (13.0)	9 (11.7)	0.81
Exercise	14 (9.1)	9 (11.7)	5 (6.5)	0.26
Others^f^	25 (16.2)	17 (22.1)	8 (10.4)	0.05

SD, standard deviation.

^a^Classified in accordance with the education system in Hong Kong.

^b^Includes experience with all types of acupuncture except abdominal acupuncture.

^c^Defined by the number of participants who used pain-relief medications over the previous week.

^d^Defined as treatment sought for neck pain at any time before this study.

^e^Includes all types of acupuncture except abdominal acupuncture.

^f^Includes bone-setting, chiropractic treatment, cupping, application of heating pad, hydrotherapy, and use of supplements.

^g^*P* values for intergroup comparison are calculated using independent two-sample *t* test or Pearson chi-square test.

**Table 2:** Primary and secondary outcome measures.

Outcome Measure	Abdominal Acupuncture (n = 77)	Sham Abdominal Acupuncture (n = 77)	Intergroup Difference	*P* Value	*P* Value^b^
**Primary outcome**					
Northwick Park Neck Pain Questionnaire scores					
Baseline	41.30 (38.22 to 44.39)	40.95 (37.61 to 44.29)		0.877^a^	
2 weeks	-11.65 (-14.39 to -8.92)	-5.90 (-8.48 to -3.33)	-5.75 (-9.48 to -2.03)	0.008^c^	
6 weeks	-11.90 (-14.62 to -9.17)	-3.25 (-5.46 to -1.04)	-8.65 (-12.13 to -5.16)	< 0.001^c^	
Overall					< 0.001
**Secondary outcomes**					
Visual analog scale scores					
Baseline	6.42 (6.09 to 6.75)	6.07 (5.66 to 6.48)		0.186^a^	
2 weeks	-2.58 (-3.01 to -2.15)	-0.79 (-1.16 to -0.43)	-1.79 (-2.35 to -1.23)	< 0.001^c^	
6 weeks	-2.36 (-2.83 to -1.89)	-0.56 (-0.93 to -0.19)	-1.80 (-2.40 to -1.21)	< 0.001^c^	
Overall					< 0.001
Short Form 36 Version 2 Health Survey questionnaire					
*Physical functioning*					
Baseline	47.37 (45.87 to 48.87)	49.39 (47.94 to 50.84)		0.056^a^	
2 weeks	1.52 (0.51 to 2.52)	-0.42 (-1.63 to 0.79)	1.94 (0.38 to 3.50)	0.045^c^	
6 weeks	2.32 (1.34 to 3.29)	-0.14 (-1.37 to 1.10)	2.45 (0.89 to 4.01)	0.007^c^	
Overall					0.003
*Role-physical*					
Baseline	42.11 (40.51 to 43.70)	44.24 (42.53 to 45.94)		0.071^a^	
2 weeks	3.15 (1.83 to 4.47)	2.16 (0.76 to 3.56)	0.99 (-0.92 to 2.90)	0.918^c^	
6 weeks	3.32 (1.94 to 4.71)	0.29 (-1.48 to 2.07)	3.03 (0.80 to 5.26)	0.024^c^	
Overall					0.012
*Bodily pain*					
Baseline	35.06 (33.78 to 36.35)	36.56 (35.30 to 37.83)		0.099^a^	
2 weeks	4.17 (2.74 to 5.60)	2.27 (0.83 to 3.71)	1.90 (-0.11 to 3.91)	0.192^c^	
6 weeks	5.86 (4.40 to 7.32)	3.00 (1.24 to 4.75)	2.86 (0.60 to 5.13)	0.041^c^	
Overall					0.022
*General health*					
Baseline	36.66 (34.81 to 38.52)	38.31 (36.29 to 40.33)		0.232^a^	
2 weeks	4.43 (3.14 to 5.73)	1.41 (-0.07 to 2.89)	3.03 (1.08 to 4.98)	0.008^c^	
6 weeks	3.50 (2.15 to 4.84)	0.23 (-1.21 to 1.67)	3.27 (1.31 to 5.22)	0.004^c^	
Overall					0.001
*Vitality*					
Baseline	43.15 (41.15 to 45.14)	43.30 (41.10 to 45.50)		0.918^a^	
2 weeks	2.74 (1.26 to 4.22)	2.43 (0.97 to 3.89)	0.31 (-1.75 to 2.37)		
6 weeks	3.59 (1.98 to 5.19)	1.89 (0.12 to 3.66)	1.70 (-0.67 to 4.07)		
Overall					0.262
*Social functioning*					
Baseline	42.24 (40.23 to 44.24)	44.97 (43.15 to 46.79)		0.046^a^	
2 weeks	3.58 (2.04 to 5.12)	1.37 (-0.30 to 3.03)	2.21 (-0.04 to 4.47)	0.162^c^	
6 weeks	3.45 (1.90 to 5.00)	0.39 (-1.24 to 2.02)	3.06 (0.83 to 5.29)	0.022^c^	
Overall					0.018
*Role-emotional*					
Baseline	41.65 (39.29 to 44.02)	44.41 (42.15 to 46.68)		0.095^a^	
2 weeks	1.99 (0.08 to 3.90)	0.45 (-1.32 to 2.23)	1.54 (-1.05 to 4.12)		
6 weeks	1.99 (0.23 to 3.75)	-1.18 (-3.33 to 0.98)	3.16 (0.41 to 5.92)		
Overall					0.054
*Mental health*					
Baseline	43.90 (42.14 to 45.67)	44.51 (42.11 to 46.92)		0.684^a^	
2 weeks	1.43 (-0.14 to 2.99)	0.37 (-1.17 to 1.92)	1.05 (-1.13 to 3.23)		
6 weeks	1.87 (0.35 to 3.38)	0.00 (-1.80 to 1.80)	1.87 (-0.47 to 4.20)		
Overall					0.227
*Physical component summary*					
Baseline	40.89 (39.55 to 42.22)	42.67 (41.41 to 43.93)		0.056^a^	
2 weeks	3.50 (2.37 to 4.63)	1.54 (0.39 to 2.68)	1.97 (0.37 to 3.56)	0.049^c^	
6 weeks	4.13 (2.99 to 5.27)	1.33 (0.12 to 2.54)	2.80 (1.15 to 4.45)	0.003^c^	
Overall					0.002
*Mental component summary*					
Baseline	42.94 (40.90 to 44.98)	44.32 (42.01 to 46.64)		0.373^a^	
2 weeks	1.97 (0.39 to 3.55)	0.83 (-0.57 to 2.22)	1.14 (-0.95 to 3.23)		
6 weeks	1.99 (0.47 to 3.52)	-0.26 (-1.95 to 1.43)	2.26 (-0.00 to 4.51)		
Overall					0.105

2 weeks: post-treatment time point; 6 weeks: 4-week post-treatment follow-up time point.

Baseline data are expressed as mean values and 95% confidence intervals.

Two and six-week data are expressed as mean improvement (and 95% confidence interval) relative to baseline values.

Intergroup differences are expressed as mean difference (and 95% confidence interval) between the abdominal and sham abdominal acupuncture groups.

^a^*P* values for intergroup comparison at baseline are calculated using independent two-sample *t* test.

^b^*P* values for group by time interaction are calculated using repeated-measures analysis of variance model.

^c^*P* values for post hoc intergroup comparison with Bonferroni correction.

**Table 3:** Credibility assessment findings.

Outcome Measure	Abdominal Acupuncture (n = 77)	Sham Abdominal Acupuncture (n = 77)	Intergroup Difference	*P* Value^a^
Improvement expected				
Baseline	3.74 (3.54 to 3.94)	3.81 (3.57 to 4.04)		0.680
2 weeks	0.40 (0.16 to 0.65)	-0.20 (-0.53 to 0.14)	0.60 (0.19 to 1.01)	0.004
Recommendation to others				
Baseline	3.87 (3.60 to 4.14)	3.73 (3.42 to 4.03)		0.482
2 weeks	0.46 (0.15 to 0.76)	-0.04 (-0.40 to 0.32)	0.49 (0.02 to 0.96)	0.039
Logical treatment				
Baseline	3.78 (3.52 to 4.04)	3.90 (3.65 to 4.14)		0.514
2 weeks	0.39 (0.11 to 0.67)	-0.09 (-0.39 to 0.21)	0.48 (0.07 to 0.89)	0.021
Effective for other complaints as well				
Baseline	3.83 (3.59 to 4.07)	3.79 (3.58 to 4.01)		0.810
2 weeks	0.27 (0.01 to 0.54)	-0.03 (-0.34 to 0.29)	0.30 (-0.11 to 0.70)	0.148

Baseline data are expressed as mean values and 95% confidence intervals.

Two-week data are expressed as mean improvement (and 95% confidence interval) relative to baseline values.

Intergroup differences are expressed as mean difference (and 95% confidence interval) between the abdominal and sham abdominal acupuncture groups.

^a^*P* values for intergroup comparison are calculated using independent two-sample *t* test.

### Primary outcome

**Fig 3:**
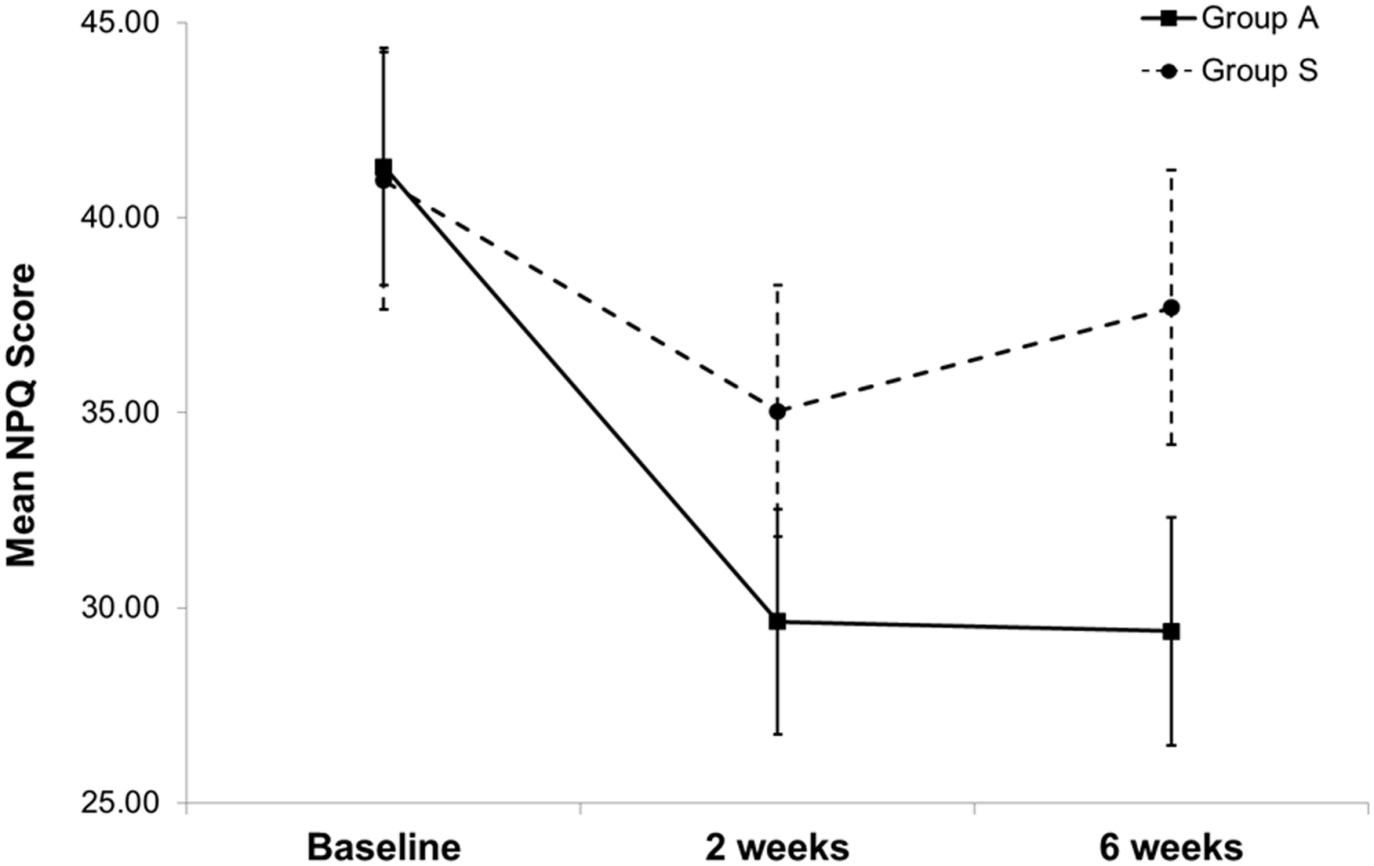
Mean Northwick Park Neck Pain Questionnaire (NPQ) scores in both groups over time. Groups A and S, patients treated by abdominal and sham abdominal acupuncture, respectively. Data are expressed as mean values and 95% confidence intervals.

The results of repeated-measures ANOVA for evaluation of changes in mean NPQ scores over time (from baseline to 2 and 6 weeks from baseline) revealed a significant group-by-time interaction (F_[2, 304]_ = 12.328; *P* < 0.001; Fig 3). At baseline, the mean NPQ scores in groups A and S were 41.30 ± 13.60 and 40.95 ± 14.73, respectively. Both groups exhibited an improvement in mean NPQ scores over time. Relative to the corresponding baseline values, the improvement in mean NPQ scores in group A was significantly greater than that in group S at both 2 weeks (intergroup mean difference, -5.75; 95% CI, -9.48 to -2.03; *P* = 0.008, after Bonferroni correction) and 6 weeks (intergroup mean difference, -8.65; 95% CI, -12.13 to -5.16; *P* < 0.001, after Bonferroni correction) from baseline (Table 2).

### Secondary outcomes

The results of repeated-measures ANOVA for evaluation of changes in mean VAS scores for neck pain intensity from baseline to 2 and 6 weeks from baseline indicated a significant group-by-time interaction (F_[1.920, 291.846]_ = 27.589; *P* < 0.001, after Greenhouse—Geisser correction). At baseline, the mean VAS scores for neck pain intensity in groups A and S were 6.42 ± 1.46 and 6.07 ± 1.82, respectively. Both groups exhibited improvements in mean VAS scores over time. Relative to the corresponding baseline values, the improvement in mean VAS scores in group A was significantly greater than that in group S at both 2 weeks (intergroup mean difference, -1.79; 95% CI, -2.35 to -1.23; *P* < 0.001, after Bonferroni correction) and 6 weeks (intergroup mean difference, -1.80; 95% CI, -2.40 to -1.21; *P* < 0.001, after Bonferroni correction) from baseline (Table 2). There was a significant difference in improvement in mean pre-post first treatment session VAS scores between groups A and S (0.98 and 0.40 points, respectively; *P* = 0.007).

The results of repeated-measures ANOVA for evaluation of changes in health-related quality of life measures over time revealed significant group-by-time interactions for physical functioning (F_[2, 304]_ = 5.849; *P* = 0.003), role-physical (F_[2, 304]_ = 4.458; *P* = 0.012), bodily pain (F_[1.891, 287.397]_ = 3.946; *P* = 0.022, after Greenhouse—Geisser correction), general health (F_[2, 304]_ = 7.331; *P* = 0.001), social functioning (F_[2, 304]_ = 4.081; *P* = 0.018), and physical component summary (F_[1.883, 286.258]_ = 6.871; *P* = 0.002, after Greenhouse—Geisser correction). Group A exhibited greater improvement in all dimensions as well as in the two SF-36v2 summary scores than group S (Table 2).

### Subsidiary observation

In general, the mean NPQ and VAS scores, and all dimensions of health-related quality of life measures for Group A continued to improve at 14 weeks relative to the corresponding baseline values. Upon inclusion of subsidiary assessment data of all primary and secondary outcome measures for group A at 14 weeks from baseline, the results of repeated-measures ANOVA revealed statistically significant differences in mean scores among different time points (baseline and 2, 6, and 14 weeks from baseline) in terms of mean NPQ (*P* < 0.001) and mean VAS (*P* < 0.001) scores as well as most domains of the SF-36v2 (S1 Table). Group A exhibited sustained improvements in these measures at 14 weeks from baseline (S1 Table).

### Other outcomes

At 2 weeks from baseline, patients in group A gave more favorable ratings for all questions on treatment credibility than did patients in group S. There were significant intergroup differences in the confidence of the participants in the treatment alleviating their complaint and in recommending the treatment to friends with similar complaints as well as in their perception of the logic of the treatment (Table 3). These results indicated that abdominal acupuncture was perceived as having higher treatment credibility than sham treatment, with the confidence in treatment being greater at 2 weeks, upon completion of the final treatment session, than at baseline.

While 85.7% of participants in group A considered the treatment to be “extremely/very/somewhat” satisfying, only 59.7% of patients in group S felt the same. On the other hand, only 1.3% of patients in group A were “somewhat/very dissatisfied” with the treatment, while 6.5% patients in group S expressed similar dissatisfaction. The rest of the participants responded with “neutral” or “no comment” when queried about treatment satisfaction.

Most participants perceived skin penetration during treatment in both groups. There were no significant intergroup differences in the findings of blinding assessment after the first treatment session (*P* = 0.066) and at 2 weeks from baseline (*P* = 0.510; Table 4).

**Table 4:** Blinding assessment findings.

	Abdominal Acupuncture (n = 77)	Sham Abdominal Acupuncture (n = 77)	*P* Value^a^
After the first treatment session			
Needle penetration	43 (55.8)	54 (70.1)	
No penetration	17 (22.1)	16 (20.8)	0.066
Don’t know	17 (22.1)	7 (9.1)	
2 weeks from baseline			
Needle penetration	63 (81.8)	63 (81.8)	
No penetration	4 (5.2)	7 (9.1)	0.510
Don’t know	10 (13.0)	7 (9.1)	

Data presented as n (%).

^a^*P* values for intergroup comparison are calculated using Pearson chi-square test.

There were no significant differences between groups A and S in the number of participants who used pain-relief medications at 2 weeks (4 [5.2%] and 10 [13.0%], respectively; *P* = 0.093) and 6 weeks (6 [7.8%] and 13 [16.9%], respectively; *P* = 0.086) from baseline.

### Adverse events

Over the 924 treatment sessions conducted for patients of both groups in this trial, 11 patients in group A developed transient bruises at the site of needle insertion one time each; they, however, did not request any medical treatment for the same (Table 5). There were no other complaints or serious adverse events in either group.

**Table 5:** Overview of adverse events.

	Abdominal Acupuncture (n = 77)	Sham Abdominal Acupuncture (n = 77)
No. of patients with any adverse events	11	0
No. of patients withdrawn due to any adverse events	0	0

## Discussion

### Main findings

This clinical trial assessed the efficacy of a 2-week standardized abdominal acupuncture treatment for neck pain. The results of this study indicated improvement in neck pain disability and pain relief in both the abdominal acupuncture and sham groups, with abdominal acupuncture being more efficacious than sham abdominal acupuncture. The improvements in NPQ, pain intensity VAS, and SF-36v2 physical component summary scores over time in the abdominal acupuncture group were all significantly greater than those in the sham treatment group. The majority of participants in group A were satisfied with abdominal acupuncture and reported no serious adverse events related to the treatment.

Interestingly, the overall ratings for treatment credibility in group A were greater than those in group S. Given that all of the included participants were abdominal acupuncture naïve, this result indicates that positive treatment effects might improve patient confidence in the treatment.

We were also able to achieve successful blinding in this study. According to a previous study, placebo needles with blunt tips are valid controls in acupuncture studies [41]. In the present study, the careful design—involving the use of needling devices of similar appearance, blindfolding of participants, and application of standardized treatment procedures in both groups—resulted in the absence of significant intergroup differences in patient perception regarding skin penetration, with most of the participants in both groups perceiving that they received abdominal acupuncture treatment and not sham treatment. Although all participants were abdominal acupuncture naïve, further analysis revealed no significant difference in the efficacy of blinding between participant with and without acupuncture experience. The success of participant blinding by means of the skin-touch sham needling method demonstrates the feasibility of blunt needles as sham devices in other clinical studies on abdominal acupuncture.

### Comparison with previous studies

A previous study provided objective evidence of the effect of abdominal acupuncture on nociceptive pathways by comparison with non-invasive placebo abdominal acupuncture [42]. The present results demonstrated that abdominal acupuncture exerted an analgesic effect and was more effective than sham abdominal acupuncture in adults suffering from neck pain. In comparison with sham treatment, abdominal acupuncture resulted in significantly greater improvements in functional performance and pain intensity at 2 and 6 weeks from the start of treatment. The reduction in mean NPQ scores from baseline in group A was over 25.0% at 2, 6, and 14 weeks from the start of treatment, indicating minimal clinically important differences [43]; in contrast group S did not exhibit clinically important differences in any of these outcome measures.

Previous studies on chronic pain have suggested that, on an average, a reduction of approximately two points on the 11-point numerical pain rating scale represents a clinically important difference [44,45]. In the present study, relative to the baseline values, group A exhibited a mean improvement of over two points on the pain VAS at all evaluation time points (2, 6, and 14 weeks from baseline).

In general, the findings of this study are consistent with those of a previous trial [24], which reported improvements in pain VAS scores after the first treatment session, upon completion of treatment, and at 3 months post-treatment among patients who received for abdominal acupuncture for treatment of cervical spondylosis. The theory of abdominal acupuncture suggests that its treatment effect is instantaneous and long lasting [12,14]. In the present study, group A exhibited significant improvements in neck pain intensity, evaluated by VAS scores, at the pre-post first treatment session as well as at the 2, 6, and 14-week evaluations.

### Strengths and limitations

This study was a meticulously designed clinical trial for evaluating whether abdominal acupuncture is more effective than non-penetrating sham abdominal acupuncture in improving functional performance and health-related quality of life and providing pain relief in adults with neck pain. We recruited participants from all regions of Hong Kong; therefore, we believe that our study participants are reasonably representative samples of local patients with neck pain. We employed validated assessment tools and ensured strict randomization and successful blinding in order to increase the reliability of outcomes. At baseline, patients of both treatment groups were homogeneous in terms of pain history and all sociodemographic characteristics and outcome measures. In addition, no attrition was noted during the study period; all participants completed the treatment and attended the follow-up evaluations, with no missing data. All these attributes significantly strengthened the validity of our findings.

However, our study has several limitations. Given the lack of rigorous research on abdominal acupuncture till date and the fact that the present study was a single-center RCT, further multicenter pragmatic trials are required to evaluate the efficacy of abdominal acupuncture in order to confirm the generalizability of the present findings in other settings. Besides, since most of our participants were women, the influence of sex on the effect of abdominal acupuncture needs further investigation. A basic set of acupuncture points were stimulated in the present study. Given that over half of the present participants had degenerative changes in the cervical spine, stimulation of more acupuncture points corresponding to the required therapeutic effect might have proved more beneficial for treatment of neck pain. In addition, the treatment method was not chosen on the basis of individual syndromes; instead, we employed a standardized protocol for all participants. Future studies taking the above limitations into consideration are required in order to explore the effect of these limitations on treatment outcomes. Furthermore, on the basis of a previous systematic review that reported six or more acupuncture treatment sessions as being associated with positive outcomes for chronic pain [46], we employed a treatment regimen of six sessions. Previous studies on abdominal acupuncture involved more treatment sessions than those employed in the present study [16,17]. Moreover, the routine abdominal acupuncture practice for neck pain usually constitutes a greater number of treatment sessions as well. Therefore, further studies are required to evaluate whether administration of treatment over a greater number of sessions could lead to greater improvement in patients with neck pain. Finally, the efficacy of abdominal acupuncture should be evaluated over a longer term than that employed in this study.

## Conclusions

Among adult patients with neck pain, abdominal acupuncture resulted in significantly greater improvements in functional performance, neck pain intensity, and some health-related quality of life measures than sham abdominal acupuncture. Abdominal acupuncture was superior to sham abdominal acupuncture in alleviating the intensity of and disability due to neck pain and improving the quality of life in patients with neck pain. These findings suggest that abdominal acupuncture is an effective treatment alternative for patients with neck pain.

## Supporting information

S1 Checklist Consolidated Standards of Reporting Trials checklist. (PDF)

S2 Checklist Standards for Reporting Interventions in Clinical Trials of Acupuncture checklist. (PDF)

S1 Protocol Study protocol. (PDF)

S1 Table Primary and secondary outcome measures over time in Group A. (PDF)
