# Supplementary material for: Efficacy of abdominal acupuncture for neck pain: A randomized controlled trial
Source: PLoS One. 2017 Jul 17;12(7):e0181360. doi: 10.1371/journal.pone.0181360 (PMC5513533; doi:10.1371/journal.pone.0181360)
Supplement: S2 Checklist — (PDF) [file pone.0181360.s002.pdf]

Checklist for items in STRICTA 2010

Item	Detail	Reported on Section
**1. Acupuncture rationale** (Explanations and examples)	1a) Style of acupuncture (e.g. Traditional Chinese Medicine, Japanese, Korean, Western medical, Five Element, ear acupuncture, etc)	Methods: Intervention
	1b) Reasoning for treatment provided, based on historical context, literature sources, and/or consensus methods, with references where appropriate	Methods: Intervention
	1c) Extent to which treatment was varied	Methods: Intervention
**2. Details of needling** (Explanations and examples)	2a) Number of needle insertions per subject per session (mean and range where relevant)	Methods: Intervention
	2b) Names (or location if no standard name) of points used (uni/bilateral)	Methods: Intervention, Fig 2
	2c) Depth of insertion, based on a specified unit of measurement, or on a particular tissue level	Methods: Intervention
	2d) Response sought (e.g. *de qi* or muscle twitch response)	Methods: Intervention
	2e) Needle stimulation (e.g. manual, electrical)	Methods: Intervention
	2f) Needle retention time	Methods: Intervention
	2g) Needle type (diameter, length, and manufacturer or material)	Methods: Intervention
**3. Treatment regimen** (Explanations and examples)	3a) Number of treatment sessions	Methods: Intervention
	3b) Frequency and duration of treatment sessions	Methods: Intervention
**4. Other components of treatment** (Explanations and examples)	4a) Details of other interventions administered to the acupuncture group (e.g. moxibustion, cupping, herbs, exercises, lifestyle advice)	Methods: Intervention
	4b) Setting and context of treatment, including instructions to practitioners, and information and explanations to patients	Methods
**5. Practitioner background** (Explanations and examples)	5) Description of participating acupuncturists (qualification or professional affiliation, years in acupuncture practice, other relevant experience)	Methods: Intervention
**6. Control or comparator interventions** (Explanations and examples)	6a) Rationale for the control or comparator in the context of the research question, with sources that justify this choice	N/A
	6b) Precise description of the control or comparator. If sham acupuncture or any other type of acupuncture-like control is used, provide details as for Items 1 to 3 above.	Methods: Intervention, Fig 2

Note: This checklist, which should be read in conjunction with the explanations of the STRICTA items, is designed to replace CONSORT 2010's item 5 when reporting an acupuncture trial.
