# Supplementary material for: Efficacy of abdominal acupuncture for neck pain: A randomized controlled trial
Source: PLoS One. 2017 Jul 17;12(7):e0181360. doi: 10.1371/journal.pone.0181360 (PMC5513533; doi:10.1371/journal.pone.0181360)
Supplement: S1 Protocol — (PDF) [file pone.0181360.s003.pdf]

A Research Proposal Entitled

Efficacy of Abdominal Acupuncture for Neck Pain: A Randomized Controlled Trial

Principal Investigator: Yuanqi Guo, School of Chinese Medicine, The Chinese University of Hong Kong

Background

Neck pain is a common and challenging health problem in the world population, with average lifetime prevalence of 48.5% [1]. It is experienced by people of all ages, with the 12-month prevalence of any neck pain among adults ranged from 12.1% to 71.5% and among children ranged from 34.5% to 71.5% [2].

In Hong Kong, neck pain was highly prevalent with increasing impact [3-4]. The 12-month prevalence of neck pain was 64.6% and around 38.0% suffered from moderate to severe pain [4]. Neck pain caused disturbances to social and work functions. There were about 17.7% of these neck pain patients had to limit their social activities and 19.0% had to limit their work [4]. Moreover, approximately 25.0% of neck pain patients have consulted medical or health professionals and it demonstrated a general trend that more neck pain patients used complementary therapies, including acupuncture [3-4].

There are different types of definitions and classification for neck pain in literature based on anatomical location, etiology, severity, and duration of symptoms [5]. According to the Bone and Joint Decade 2000-2010 Task Force on Neck Pain and Its Associated Disorders, neck pain was specific to pain located in the anatomic region of the neck, with or without radiation to the head, trunk and upper limbs [6]. The

anatomic region of the neck from the back referred to the region from the superior nuchal line to the spine of the scapula and the anatomic region of the neck from the side referred to the region from the superior nuchal line down to the superior border of the clavicle and the suprasternal notch.

There are various invasive and noninvasive treatment options available for patients with neck pain, including medications, injection, manual therapies, physical modalities, complementary and alternative medicine therapies, education or advice, and surgery [7-8]. However, due to the paucity of evidence from primary studies, the efficacy of currently available treatments for neck pain remains unclear [9-10].

Abdominal acupuncture is a therapeutic intervention based on traditional Chinese medicine acupuncture meridian theory and new concepts developed by Dr Zhiyun Bo after decades of research [11]. According to abdominal acupuncture, acupuncture needles are used to stimulate the abdominal meridian system. The abdominal meridian system is a network of meridian and acupuncture points governed by the *shenque* system. It is based on the idea that, the acupuncture point Shenque (CV8) formed the foundation of all regulating systems of the body at the embryological stage, regulating the distribution of qi and blood to the whole body [12]. By puncturing specific acupuncture points on the abdomen, the system can be used to restore harmony and achieve a holistic balance of the body. There are three levels of needle penetration in abdominal acupuncture with distinctive functions and therapeutic effects, in which deep needling is used to regulate the viscera and bowels; medium needling is used to regulate the meridians and shallow needling is used to treat symptoms. During abdominal acupuncture treatment, the skin tissue is needled through its superficial layers. With the needles insertion mainly on the superficial skin, abdominal fat down

to the superficial layer of abdominal muscle, abdominal acupuncture is safe and no injury would be found on the abdominal organs. The treatment is rather painless and more acceptable to patients as needle penetration is much shallower than conventional techniques and obtaining qi will not be elicited. Abdominal acupuncture can be used in treatment of various disorders, including in particular pain and neurological disorders. By concentrating acupuncture on specific points and areas on the abdominal region, all parts of the body can be treated as the energy of the whole body is connected [11-14].

Previous reviews and studies on acupuncture treatments have shown positive effect on pain relief for various disorders, including headache, neck and shoulder pain and back pain [15-19]. As regards to neck pain, the results from two systematic reviews on randomized controlled trials showed that abdominal acupuncture had positive effect for treatment of neck pain, including pain relief in terms of improvements on visual analogue scale (VAS) on pain, pain rating index (PRI) and present pain intensity (PPI) by using the McGill pain questionnaire [20-21]. However, due to the lack of large sample size, high quality and well-designed studies, its effectiveness is still inconclusive [16,19-21].

In view of the importance of the health problem of neck pain, a carefully designed randomized, placebo-controlled, patient and assessor-blind clinical trial is proposed.

Objectives

This study aims to evaluate the efficacy of abdominal acupuncture for the treatment of neck pain. It will examine whether abdominal acupuncture treatment can reduce neck pain disability and intensity, and improve quality of life for neck pain patients.

Methods

Study design

This study is a single center, patient and assessor-blind, placebo-controlled, parallel-group study with balanced randomization that compares standardized abdominal acupuncture with controlled non-penetrating sham abdominal acupuncture. The trial will be conducted at Pok Oi Hospital – The Chinese University of Hong Kong Chinese Medicine Centre for Teaching and Research (Shatin). Clinical ethics approval will be obtained from the Joint CUHK–NTEC Clinical Research Ethics Review Committee before the trial starts. Eligible participants are blinded to treatment group allocation. The participants will be told before randomization that they are allocated to one of two groups. They will be informed that the treatment might or might not prove to be effective and that there will be a 50% chance of assigning to a sham group but do not tell what the sham treatment is. The outcome assessment and statistical analysis will be performed by a research assistant who is also blinded to patient assignment.

Participants

This study will focus on patients aged between 18 and 65 years with neck pain. Entry inclusion and exclusion criteria are developed mainly to enroll appropriate participants and exclude those with complicated neck pain or having serious diseases.

Inclusion criteria

Patients will be included in the study if they satisfied the following criteria:

- between 18 and 65 years of age with the ability to speak and understand Cantonese;

- neck complaints on:
    - pain, stiffness or tenderness, with or without musculoskeletal signs including decreased range of motion and point tenderness [22];
    - pain felt in the neck with radiation to the occiput or towards the shoulders, or both with limitation of neck motion [22];
    - neck pain with degenerative joint disease or cervical spondylosis, or both [22];
- 3 points or more on a 10-cm visual analogue scale for neck pain at the time of screening;
- no previous treatment with abdominal acupuncture for any reason;
- agreed to be randomized; and
- agreed to sign the informed consent form.

Exclusion criteria

Patients will be excluded from the study if they possess any of the following criteria:

- visceral pain in the neck, including carcinoma of thyroid or larynx and tuberculosis of larynx [22];
- serious spinal disorders, including malignancy, vertebral fracture, spinal infection and inflammatory spondylitis;
- patients who previously had neck surgery or are scheduled to have one during the study;
- other chronic diseases that could interfere with abdominal acupuncture treatment effects, including cardiovascular disease, diabetic neuropathy, active hepatitis, fibromyalgia, rheumatoid arthritis, dementia and epilepsy;
- diagnosis of cancer of any nature;
- chief musculoskeletal pain other than neck pain;

- conditions that abdominal acupuncture might not be safe, including clotting disorders, administration of an anticoagulant agent, acute abdominal disorders, venous dilation of the naval, celiac tumor and pregnant women;
- possess abdominal scar which will affect the proper selection of acupuncture points for abdominal acupuncture;
- severe psychiatric or psychological disorder;
- acupuncture treatment for neck pain during the previous month;
- conflicting or ongoing co-interventions (drugs or alternative treatments, or both);
- engage in any other clinical trial during the study period;
- pending neck-related litigation or disability claims;
- incapacity to complete the questionnaires or to answer the questions of the assessor in the study; and
- pregnant and breast feeding women.

Recruitment

Participants will be recruited through advertisements or posters posted on bulletin boards of all Chinese medicine centers, polyclinics and mobile clinics under Pok Oi Hospital and leaflets delivered through these sites. Patients interested in participating in the study will be referred to Pok Oi Hospital – The Chinese University of Hong Kong Chinese Medicine Centre for Teaching and Research (Shatin) to undergo a process to determine eligibility. They will be first screened and checked by Hong Kong Registered Chinese Medicine Practitioners (RCMPs) who have at least 3 years clinical experience and have received pre-study training by a senior RCMP. The RCMPs will run down the checklist of inclusion and exclusion criteria. A full medical history will be obtained, as well as clinical examination and cervical radiography. The clinical examination consist of inspection of the neck and shoulder regions for muscle

atrophy and dislocations, active and passive movements of neck and shoulder joints and provocation tests of neck and shoulder muscles. Those suitable to be recruited to the study will be asked to see the research assistant of the center. Then, the research assistant will explain the overall objectives and nature of the study and guides them through the informed consent process. The research assistant will administer the baseline questionnaire and written informed consent. This is followed by random allocation to real abdominal acupuncture group or sham abdominal acupuncture group by an administration staff of the Chinese medicine center. After randomization, the research assistant will schedule treatment procedure. The study flow is shown in Figure 1.

Figure 1. The study flow of abdominal acupuncture for neck pain

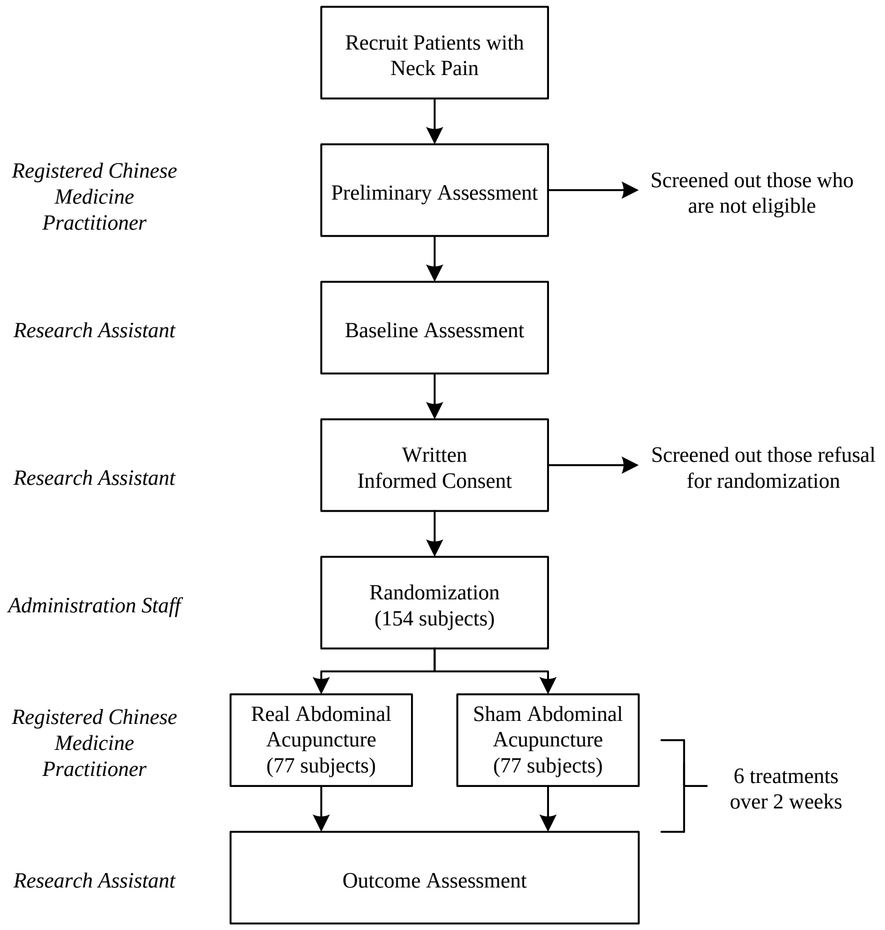

Randomization

Treatment allocation of real abdominal acupuncture or sham abdominal acupuncture for each eligible patient in the study will be determined by a randomization process. The random allocation to the 2 arms of the study will be performed using a computer software named Random Allocation Software [23], following a balanced 1:1 pattern (real abdominal acupuncture:sham abdominal acupuncture) based on a block design where the block size is not constant. This will be carried out by an administration staff of the centre who is not involved in the study and the information of the allocation list will remain strictly confidential. All participants, screeners, practitioners and outcome assessors involving in this study are masked to the whole randomization process. Random assignments cannot be viewed in advance nor changed after randomization. The specific treatments patients are assigned to are only revealed to the practitioners immediately prior to treatment.

Background of practitioners

The treatments of the study will be performed by RCMPs who have over 3 years experience in acupuncture and possess specialty in administering abdominal acupuncture. The RCMPs in this study should strictly adhere to the study protocol and trained to be familiar with administering study treatments. Besides, they should have full understanding of the procedure to carry out sham abdominal acupuncture. Furthermore, they should agree to accept the allocation of treatment and would not deviate, treat each patient to the best of their professional skills and attention for all study subjects. The practitioners will be asked to maintain the same attitude to both treatment groups to avoid any psychological influences.

Treatment details

The abdominal acupuncture, a therapeutic microsystem based on traditional Chinese medicine acupuncture meridian theory and new concepts developed by Dr Zhiyun Bo [11-14], will be used for acupuncture point selection and administration in this study. Patients will undergo a total of 6 treatments as previous articles or reviews have shown that six or more acupuncture treatments were significantly associated with positive outcomes [24-25]. Both real and sham abdominal acupuncture groups will be treated every other day, three times a week for 2 weeks (6 treatments in total). The treatments will be provided at no cost to all participants. Electrostimulation, moxibustion, cupping, tuina, and any other non-needle treatments and adjuncts will not be allowed in this study. Any adverse reactions or collateral effects will be recorded with detailed explanation of their type and the dates on which they occurred.

Real abdominal acupuncture treatment

In the real treatment group, a pre-defined standardized abdominal acupuncture prescription that has been used in previous studies for neck pain will be employed [11, 15,17]. The acupuncture points used will be: Zhongwan (CV12), Guanyuan (CV4), bilateral Shangqu (KI17) and bilateral Huaroumen (ST24) and they are shown in Figure 2. Abdominal acupuncture treatments will be provided using sterile, single-use, disposable Bo’s abdominal acupuncture needles (40 mm x 0.22 mm) (Changzi City Enyi Science and Technology Co., Ltd, China) with the aid of guide tubes for inserting needles. The patient wearing eye mask will be lying in a supine position and the abdomen is exposed. The abdomen is examined for any contraindication to abdominal acupuncture and the skin will be sterilized before needle insertion. Then, the location of the Conception Vessel is defined. Each treatment begins with needle insertion in the acupuncture points Zhongwan (CV12) and Guanyuan (CV4) for

invigorating the spleen and tonifying the kidney of the body, followed by the rest of the acupuncture points. The abdominal acupuncture treatment will include three steps, namely, awaiting qi, move qi and hastening qi. The first step is awaiting qi. It works by means of inserting the needles perpendicularly with the use of guide tubes to superficial level of skin for all the selected acupuncture points. After 3 to 5 minutes, it will progress to the step of move qi. This will be done by inserting the needles perpendicularly in the acupuncture points Zhongwan (CV12) and Guanyuan (CV4) deeply down to superficial level of the abdominal muscle (deep needling, to a depth of 20–35 mm), bilateral Shangqu (KI17) to the superficial layer of abdomen (shallow needling, to a depth of 5 mm) and inserting the needles for bilateral Huaroumen (ST24) down to subcutaneous abdominal fat (medium needling, to a depth of 10–20 mm). Then, it is followed by hastening qi by making appropriate adjustment on the depth of the needles according to pain relief condition of patients. The manipulation method is light and only minimal twirling is required. Responses such as obtaining qi or muscle twitch responses will not be sought. The needles will be retained for 30 minutes with an infrared therapeutic lamp (Chongqing Xinfeng Medical Instruments Co., Ltd, China) being placed 30 cm directly over the navel. After finishing the needles retention time, the needles will be removed following the sequence of acupuncture point insertion. Each insertion hole will be pressed for a while with a dry sterilized cotton ball immediately after withdrawal of the needle following the open-closed supplementation and draining method.

Figure 2. The acupuncture points used in the study

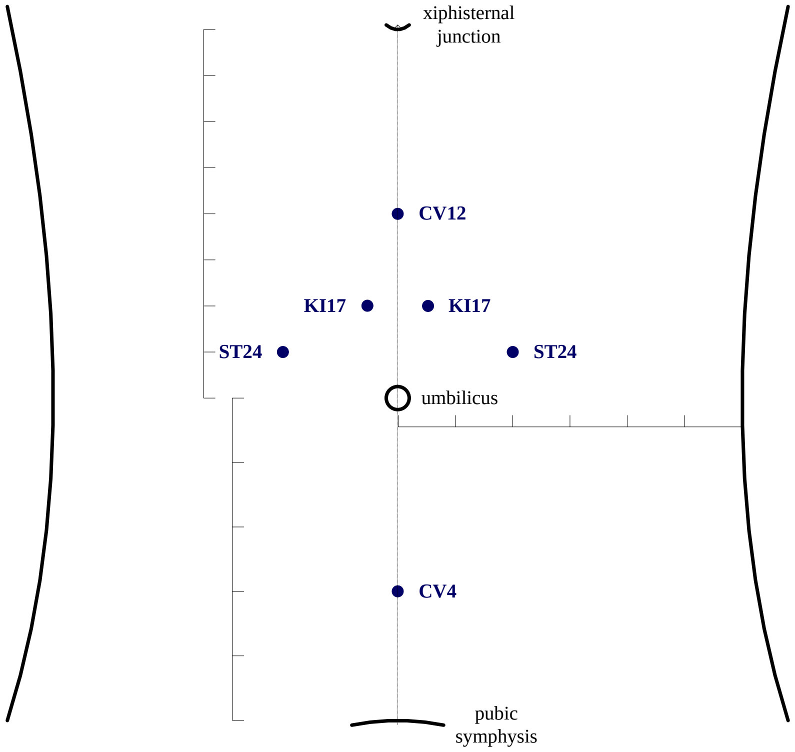

Sham abdominal acupuncture treatment

In the sham treatment group, single-use, disposable non-penetrating blunt sham needles with guide tubes will be used. The acupuncture points used will be 1 *cun* away from the points used in the real abdominal acupuncture group. After the skin sterilization, the RCMP will mimicked the real abdominal acupuncture treatment by using the blunt sham needle with the patient lying in a supine position and wearing eye mask. The RCMP will pretend to insert needle using guide tubes similar to real abdominal acupuncture treatment. When the needle is pressed on the skin, the blunted tip will touch the skin, giving patient feeling of real needle insertion. The treatment procedure and techniques used in this group will be the same as the real treatment group. An infrared therapeutic lamp (Chongqing Xinfeng Medical Instruments Co., Ltd, China) will be placed 30 cm directly over the navel for the duration of the

30-minute retention time in each treatment session.

After the completion of the 4-week follow-up, the patients randomized to this group will receive compensation treatments of real abdominal acupuncture as described in the real treatment group.

Throughout all the treatment sessions for both real and sham abdominal acupuncture groups, a screen and eye masks will be used to obstruct patients’ sight from seeing the needles.

Co-interventions

During the study period which includes the follow-up period of the study, each participant is advised by the practitioner to avoid other treatments such as physiotherapy, acupressure, cupping, tuina, reflexotherapy, chiropractic or bone-setting for neck pain. This will be reinforced by the research assistant during the entry period and at each evaluation time point. In the event that a patient has attended other co-interventions for neck pain, including using pain relief medications, it should be reported to the research assistant during each evaluation period and the information will be recorded by the research assistant. However, general light exercises are allowed for all patients participating in the study.

Ethical considerations

This study will adhere to the common guidelines of the Declaration of Helsinki for medical research involving human subjects [26-27] and the International Conference on Harmonisation – Good Clinical Practice Guidelines [28]. Prior ethical approval will be obtained from the Joint CUHK–NTEC Clinical Research Ethics Review

Committee before the commencement of the study. Prior verbal and written consent will be obtained from each patient participating in the study.

Stopping rules

The study will be stopped if the principal investigator believes that there is an unacceptable risk of serious adverse event in one or more of the treatment groups.

Outcome measurements

Baseline information

Sociodemographic information of all participants, including age, gender, education background and neck pain history including pain sites (unilateral neck, bilateral neck, central neck), pain duration, pain occurrence, previous neck injury, and previous neck treatments, will be recorded at baseline interview. An outcome assessor who is masked to treatment allocation will conduct assessments.

The study's primary endpoint will be immediately after the intervention ends. Table 1 below summarizes the categories of information and measurements that are included in the baseline and follow-up interviews.

Table 1. Content of baseline and follow-up assessments

**Measures**		**Baseline**	**Finished**	**4-week**	**12-week**
Inclusion and exclusion variables		X			
Sociodemographic characteristics		X			
Neck pain history		X			
Neck Pain Questionnaire	- Real	X	X	X	X
	- Sham	X	X	X	
VAS for pain intensity†	- Real	X	X	X	X
	- Sham	X	X	X	
SF-36v2 Health Survey	- Real	X	X	X	X
	- Sham	X	X	X	
Credibility test		X	X		
Satisfaction with treatment			X		
Blinding assessment†			X		
Use of medications*		X	X	X	X
Adverse experiences*			X	X	X
Dropouts and withdrawals*		X	X	X	X

*At every single visit; †After the first treatment

Primary outcome measure

The primary outcome measure is the Northwick Park Neck Pain Questionnaire (NPQ) [29]. It is a questionnaire used to evaluate functional performance in patients with neck pain [5]. In this study, the validated Chinese version of the NPQ will be used [30-32]. It consists of nine five-part questions to assess the patient's symptoms and a score is obtained [29]. The questions cover activities which are likely to be affected

by neck pain. Each question contains five statements of increasing difficulty and is scored between 0 to 4. The NPQ will be scored out of 36, or 32 for non-car drivers, and is presented as a percentage [29,33]. In this study, the questionnaire will be assessed at baseline, immediately after the intervention ends, at 4-week, and 12-week follow-up interviews for real abdominal acupuncture group. As for sham abdominal acupuncture group, the questionnaire will be assessed at baseline, immediately after the intervention ends, and at 4-week follow-up interview.

Secondary outcome measures

The visual analogue scale (VAS) for pain is a measurement of pain intensity and has widely used [5,34]. This is a continuous scale of 10-cm in length. Participants will be asked to mark on the scale 0 (no pain) to 10 (worst imaginable pain), the degree of intensity of neck pain experienced on the day the assessment is performed. This item will be assessed at baseline, after the first treatment, immediately after the intervention ends, at 4-week, and 12-week follow-up interviews for real abdominal acupuncture group. As for sham abdominal acupuncture group, it will be assessed at baseline, after the first treatment, immediately after the intervention ends, and at 4-week follow-up interview.

The SF-36 Health Survey is a generic questionnaire that used to measure the general health status, including physical and mental components, with higher scores indicate better health [5,35]. In this study, the validated Chinese (HK) SF-36v2 Health Survey will be used [36]. This measure will be assessed at baseline, immediately after the intervention ends, at 4-week, and 12-week follow-up interviews for real abdominal acupuncture group. As for sham abdominal acupuncture group, it will be assessed at baseline, immediately after the intervention ends, and at 4-week follow-up interview.

Other measures

The well-validated credibility scale developed by Borkovec and Nau [37] which has previously been used for acupuncture studies, will be used to ascertain treatment credibility in the two groups [35,38-39]. All participants will be asked, “How confident do you feel that this treatment can alleviate your complaint?”, “How confident would you be in recommending this treatment to a friend who suffered from similar complaints?”, “How logical does this treatment seem to you?” and “How successful do you think this treatment would be in alleviating other complaints?” at baseline and immediately after the intervention ends, scoring on a 0 to 6 Likert scale, with 6 indicating higher credibility [35].

A global measure of satisfaction with care and treatment outcome will also be evaluated immediately after the intervention ends (the end of 6 treatments). All patients will need to answer the question: “All things considered, how satisfied are you with the results of your recent treatment for neck pain?” (1 = extremely satisfied, 2 = very satisfied, 3 = somewhat satisfied, 4 = mixed (approximately equal satisfaction and dissatisfaction), 5 = somewhat dissatisfied, 6 = very dissatisfied, 7 = extremely dissatisfied, 8 = not sure/no opinion) [40].

Blinding assessments will be evaluated after the first treatment and immediately after the intervention ends to test blinding to the two treatment groups. Patients in both real and sham abdominal acupuncture groups will be asked to guess whether they are in the real treatment group or the sham control group. Besides, in order to examine the efficacy of the blinding technique of this study, the subjects will be asked to select an answer for the question “How did you feel when the acupuncture needle was inserted?”

The available answers are: (1) needles were inserted into the muscle; (2) needles did not penetrate the skin; and (3) I could not discriminate the difference [41].

Patients' use of pain relief medications during the study period will be recorded throughout the study period.

Adverse events

Any complaints and symptoms, including pain, skin irritations, bruise at acupuncture points, subcutaneous bleeding, dizziness, and any other unusual responses will be monitored and recorded throughout the whole study period.

Dropouts and withdrawals

All dropouts and attrition during the course of the study will be monitored and recorded with respective reasons.

Statistical issues

Sample size determination

The sample size will be determined with a general acceptance of probability of 0.05 in committing a type I error and a power of 80%. For two arms (control — sham abdominal acupuncture and experimental — real abdominal acupuncture), repeated measures of analysis of variance (ANOVA) model will be used. An effect size of 0.2, which considered as small by Cohen [42], will be adopted. By using the power analysis program G*Power 3 [43] with consideration of equal allocation between the two groups and a dropout rate of 15%, it is estimated that each group will require 77 subjects, with a total sample size of 154 subjects for this study.

Statistical methods and analysis

Analyses of data in this study will be performed by using statistical analysis software (SPSS 20.0). Two-sided significance will be defined as $P < 0.05$.

All the demographic, clinical, baseline and adverse events data will be analyzed descriptively, using means and standard deviations and 95% confidence intervals (CIs) for the continuous variables and percentages for the categorized ones. These data will be performed separately for each study group. Baseline characteristics of all study groups will be compared to examine homogeneity. Independent two-sample t-test will be used to determine possible baseline differences for quantitative data. The Chi-square test will be used to determine possible differences for qualitative data.

An intention-to-treat approach will be used for all analyses in this study for all the randomized patients to assess the efficacy of abdominal acupuncture treatment. Missing data will be replaced with the last value obtained from the patients, and such value will be carried forward if no additional value obtained. This approach is based on the assumption that patients who have missing values do not have improvement since the last follow-up assessment and may expect no further benefit from the therapy.

Repeated measures ANOVA will be used as single overall tests for the effects of treatment and for the comparison between real and sham abdominal acupuncture groups at baseline, immediately after the intervention ends, and at 4-week follow-up interviews. The ANOVA will be performed for each outcome variable separately. Between groups effects of measurement for each outcome variable at different assessments will be analyzed by ANOVA while the difference of outcomes between

real and sham abdominal acupuncture groups will be assessed by the independent sample t-test.

Conclusion

This is the first rigorously designed evidence-based clinical trial to evaluate whether abdominal acupuncture is more efficacious in relieving pain than non-penetrating sham abdominal acupuncture in patients with neck pain in Hong Kong. The results obtained in the proposed study would provide valuable information about the efficacy of abdominal acupuncture for neck pain and set a solid foundation for future full-scale clinical study on the same topic. The development of the abdominal acupuncture protocol through evidence-based approach would be very important in providing an efficacious alternative treatment method for further clinical management of neck pain.

References

1. Fejer R, Kyvik KO, Hartvigsen J. The prevalence of neck pain in the world population: a systematic critical review of the literature. *European Spine Journal* 2006;15(6):834-48.

2. Hogg-Johnson S, van der Velde G, Carroll LJ, Holm LW, Cassidy JD, Guzman J, et al. The burden and determinants of neck pain in the general population: results of the Bone and Joint Decade 2000-2010 Task Force on Neck Pain and Its Associated Disorders. *Spine* 2008;33(4 Suppl):S39-51.

3. Chiu TT, Leung AS. Neck pain in Hong Kong: a telephone survey on prevalence, consequences, and risk groups. *Spine* 2006;31(16):E540-4.

4. Chiu TT, Leung AS, Lam P. Neck pain in Hong Kong: a telephone survey on consequences and health service utilization. *Spine* 2010;35(21):E1088-95.

5. Misailidou V, Malliou P, Beneka A, Karagiannidis A, Godolias G. Assessment of patients with neck pain: a review of definitions, selection criteria, and measurement tools. *Journal of Chiropractic Medicine* 2010;9(2):49-59.

6. Guzman J, Hurwitz EL, Carroll LJ, Haldeman S, Côté P, Carragee EJ, et al. A new conceptual model of neck pain: linking onset, course, and care: the Bone and Joint Decade 2000-2010 Task Force on Neck Pain and Its Associated Disorders. *Spine* 2008;33(4 Suppl):S14-23.

7. Carragee EJ, Hurwitz EL, Cheng I, Carroll LJ, Nordin M, Guzman J, et al.

Treatment of neck pain: injections and surgical interventions: results of the Bone and Joint Decade 2000-2010 Task Force on Neck Pain and Its Associated Disorders. *Spine* 2008;33(4 Suppl):S153-69.

8. Hurwitz EL, Carragee EJ, van der Velde G, Carroll LJ, Nordin M, Guzman J, et al. Treatment of neck pain: noninvasive interventions: results of the Bone and Joint Decade 2000-2010 Task Force on Neck Pain and Its Associated Disorders. *Spine* 2008;33(4 Suppl):S123-52.

9. Aker PD, Gross AR, Goldsmith CH, Peloso P. Conservative management of mechanical neck pain: systematic overview and meta-analysis. *British Medical Journal* 1996;313(7068):1291-6.

10. Hoving JL, Gross AR, Gasner D, Kay T, Kennedy C, Hondras MA, et al. A critical appraisal of review articles on the effectiveness of conservative treatment for neck pain. *Spine* 2001;26(2):196-205.

11. Bo Z. *Abdominal Acupuncture*. Beijing: Science & Technology Publishing Press, 1999.

12. Bo Z. The Shenque system and abdominal acupuncture. *Beijing Journal of Traditional Chinese Medicine* 1993;4:13-4.

13. Lore R. Abdominal acupuncture: a practical introduction. *Journal of Chinese Medicine* 2007;83:29-32.

14. Scott T. Abdominal acupuncture (fu zhen): energetic and clinical applications. *Journal of Chinese Medicine* 2008;87:16-8.

15. Bo Z, Niu Q, Zhu W, Xiang Y, Wang G, Yuan S. Multicenter controlled study on abdominal acupuncture for treatment of nerve root type cervical spondylosis. *Chinese Acupuncture & Moxibustion* 2005;25(6):387-9.

16. Trinh K, Graham N, Gross A, Goldsmith CH, Wang E, Cameron ID, et al. Acupuncture for neck disorders. *Cochrane Database of Systematic Reviews* 2006, Issue 3. Art No.: CD004870. DOI:10.1002/14651858.CD004870.pub3.

17. Guo Y, Chen L, Fu W, Xu M, Ou X. Clinically randomized controlled study on abdominal acupuncture for treatment of cervical spondylosis. *Chinese Acupuncture & Moxibustion* 2007;27(9):652-6.

18. Wang S, Xu M, Guo Y. Advances of clinical studies on abdominal acupuncture for treatment of pain diseases. *Journal of New Chinese Medicine* 2009;41(11):106-8.

19. Ernst E, Lee MS, Choi T. Acupuncture: does it alleviate pain and are there serious risks? A review of reviews. *Pain* 2011;152:755-64.

20. Wang Y, Fu W, Ou A, Fan L, Huang Y. A systematic review of randomized controlled clinical trials of abdominal acupuncture treatment of cervical spondylosis. *Acupuncture Research* 2011;36(2):137-44.

21. Yang L, Fu W, Zhang G, Huang Y, Zhang M. Systematic review on effect of

abdominal acupuncture for cervical spondylosis. *China Journal of Traditional Chinese Medicine and Pharmacy* 2012;27(2):319-23.

22. Zhu XM, Polus B. A controlled trial on acupuncture for chronic neck pain. *The American Journal of Chinese Medicine* 2002;30(1):13-28.

23. Saghaei M. Random allocation software for parallel group randomized trials. *BMC Medical Research Methodology* 2004;4:26.

24. Ezzo J, Berman B, Hadhazy VA, Jadad AR, Lao L, Singh BB. Is acupuncture effective for the treatment of chronic pain? A systematic review. *Pain* 2000;86(3):217-25.

25. White AR, Filshie J, Cummings TM. Clinical trials of acupuncture: consensus recommendations for optimal treatment, sham controls and blinding. *Complementary Therapies in Medicine* 2001;9(4):237-45.

26. World Medical Association. WMA Declaration of Helsinki – ethical principles for medical research involving human subjects (October 2008). http://www.wma.net/en/30publications/10policies/b3/index.html (accessed 25 August 2012).

27. Horner D, Foëx BA. The ethics of clinical trials. *Anaesthesia and Intensive Care Medicine* 2011;13(1):7-10.

28. International Conference on Harmonisation. Guideline for Good Clinical Practice

E6(R1) (1996). http://www.ich.org/products/guidelines/efficacy/efficacy-single/article/good-clinical-practice.html (accessed on 17 January 2014).

29. Leak AM, Cooper J, Dyer S, Williams KA, Turner-Stokes L, Frank AO. The Northwick Park Neck Pain Questionnaire, devised to measure neck pain and disability. *British Journal of Rheumatology* 1994;33(5):469-74.

30. Chiu TT, Lam TH, Hedley AJ. Subjective health measure used on Chinese patients with neck pain in Hong Kong. *Spine* 2001;26(17):1884-9.

31. Chiu TT, Lam TH, Hedley AJ. A randomized controlled trial on the efficacy of exercise for patients with chronic neck pain. *Spine* 2005;30(1):E1-7.

32. Chiu TT, Ng JK, Walther-Zhang B, Lin RJ, Ortelli L, Chua SK. A randomized controlled trial on the efficacy of intermittent cervical traction for patients with chronic neck pain. *Clinical Rehabilitation* 2011;25(9):814-22.

33. Salter GC, Roman M, Bland MJ, MacPherson H. Acupuncture for chronic neck pain: a pilot for a randomized controlled trial. *BMC Musculoskeletal Disorders* 2006;7:99.

34. Hawker GA, Mian S, Kendzerska T, French M. Measures of adult pain. *Arthritis Care & Research* 2011;13(S11):S240-52.

35. White P, Lewith G, Prescott P, Conway J. Acupuncture versus placebo for the

treatment of chronic mechanical neck pain: a randomized, controlled trial. *Annals of Internal Medicine* 2004;141(12):911-9.

36. Lam ETP, Lam CLK, Lo YYC, Gandek B. Psychometrics and population norm of the Chinese (HK) SF-36 Health Survey_Version 2. *The Hong Kong Practitioner* 2008;30(4):185-98.

37. Borkovec TD, Nau SD. Credibility of analogue therapy rationales. *Journal of Behavior Therapy and Experimental Psychiatry* 1972;3(4):257-60.

38. Vincent C, Lewith G. Placebo controls for acupuncture studies. *Journal of the Royal Society of Medicine* 1995;88(4):199-202.

39. Zhang SP, Yip TP, Li QS. Acupuncture treatment for plantar fasciitis: a randomized controlled trial with six months follow-up. *Evidence-Based Complementary and Alternative Medicine* 2011; Article ID 154108, 10 pages, 2011. doi:10.1093/ecam/nep186.

40. Hudak PL, Wright JG. The characteristics of patient satisfaction measures. *Spine* 2000;25(24):3167-77.

41. Itoh K, Katsumi Y, Hirota S, Kitakoji H. Randomised trial of trigger point acupuncture compared with other acupuncture for treatment of chronic neck pain. *Complementary Therapies in Medicine* 2007;15(3):172-9.

42. Cohen J. *Statistical Power Analysis for the Behavioral Sciences*. 2nd edition.

Hillsdale, NJ: Lawrence Erlbaum Associates, 1998.

43. Faul F, Erdfelder E, Lang A-G, Buchner A. G*Power 3: a flexible statistical power analysis program for the social, behavioral, and biomedical sciences. *Behavior Research Methods* 2007;39(2):175-91.
