# Supplementary material for: Efficacy of abdominal acupuncture for neck pain: A randomized controlled trial
Source: PLoS One. 2017 Jul 17;12(7):e0181360. doi: 10.1371/journal.pone.0181360 (PMC5513533; doi:10.1371/journal.pone.0181360)
Supplement: S1 Table — (PDF) [file pone.0181360.s004.pdf]

S1 Table. Primary and secondary outcome measures over time in Group A.

	Baseline	2 weeks	6 weeks	14 weeks	*F* Value[a]	*P* Value[a]
**Primary Outcome**						
NPQ scores	41.30 (38.22 to 44.39)	29.65 (26.72 to 32.58)[b]	29.41 (26.43 to 32.38)[b]	29.38 (26.17 to 32.59)[b]	40.95	< 0.001
**Secondary Outcomes**						
Pain VAS scores	6.42 (6.09 to 6.75)	3.84 (3.37 to 4.31)[b]	4.06 (3.53 to 4.60)[b]	4.20 (3.65 to 4.75)[b]	58.20	< 0.001
SF-36v2 Health Survey						
Physical functioning	47.37 (45.87 to 48.87)	48.89 (47.62 to 50.17)[b]	49.69 (48.32 to 51.05)[b]	50.09 (48.70 to 51.47)[b]	12.46	< 0.001
Role-physical	42.11 (40.51 to 43.70)	45.26 (43.66 to 46.86)[b]	45.43 (43.82 to 47.04)[b]	44.67 (43.00 to 46.35)[b]	9.76	< 0.001
Bodily pain	35.06 (33.78 to 36.35)	39.23 (37.92 to 40.54)[b]	40.92 (39.45 to 42.39)[b]	41.23 (39.55 to 42.90)[b]	30.90	< 0.001
General health	36.66 (34.81 to 38.52)	41.10 (39.15 to 43.04)[b]	40.16 (38.19 to 42.12)[b]	40.77 (38.71 to 42.84)[b]	18.41	< 0.001
Vitality	43.15 (41.15 to 45.14)	45.89 (44.00 to 47.77)[b]	46.74 (44.58 to 48.89)[b]	45.92 (43.69 to 48.16)[b]	7.19	< 0.001
Social functioning	42.24 (40.23 to 44.24)	45.82 (43.97 to 47.66)[b]	45.69 (43.80 to 47.57)[b]	46.66 (44.94 to 48.39)[b]	14.15	< 0.001
Role-emotional	41.65 (39.29 to 44.02)	43.64 (41.81 to 45.47)	43.64 (41.74 to 45.54)	44.05 (42.06 to 46.04)	2.79	0.046
Mental health	43.90 (42.14 to 45.67)	45.33 (43.40 to 47.26)	45.77 (43.83 to 47.72)	44.48 (42.57 to 46.39)	2.43	0.066
Physical component summary	40.89 (39.55 to 42.22)	44.39 (43.10 to 45.68)[b]	45.02 (43.62 to 46.41)[b]	45.35 (43.88 to 46.81)[b]	31.56	< 0.001
Mental component summary	42.94 (40.90 to 44.98)	44.91 (43.00 to 46.81)	44.93 (42.91 to 46.95)	44.52 (42.50 to 46.53)	2.94	0.039

Baseline, 2, 6, and 14-week data are expressed as mean values and 95% confidence intervals.

[a]*F* values and *P* values are calculated using one-way repeated-measures analysis of variance model.

[b]Post hoc pairwise comparisons with Bonferroni correction showed statistically significant effect as compared with baseline ($P < 0.05$).
